# The complete mitochondrial genome of the silkworm, *Bombyx mori* strain BaiyuN

**DOI:** 10.1080/23802359.2018.1443039

**Published:** 2018-02-26

**Authors:** Gang Li, Heying Qian, Guodong Zhao, Anying Xu

**Affiliations:** a Jiangsu University of Science and Technolgy, Zhenjiang, Jiangsu, China;; b Key Laboratory of Silkworm and Mulberry Genetic Improvement, Ministry of Agriculture, Jiangsu University of Science and Technology, Zhenjiang, Jiangsu, China

**Keywords:** *Bombyx mori* nucleopolyhedrovirus, mitogenome, next-generation sequencing

## Abstract

Here, we report the complete mitochondrial genome of a *Bombyx mori* strain BaiyuN, which is identified to be highly resistant to *Bombyx mori* nucleopolyhedrovirus (BmNPV). Its complete mitochondrial genome is 15,655 bp in length (GenBank accession no. MG797555), consisting of 13 protein-coding genes, 22 tRNA genes, 2 rRNA, and 1 control region (494 bp). The complete mitogenome of the *B. mori* strain BaiyuN could provide a basic data for further phylogenetics and antivirus research.

The silkworm, *Bombyx mori*, holometabolous insect, is a key model of the Lepidoptera (Xia et al. 2009). BaiyuN is one strain, which was collected from Zhenjiang city, Jiangsu Province in China (N 32.20’, E 119.44’). Samples have been conserved in the national centre for silkworm genetic resources preservation, Chinese academy of agricultural sciences (SGRP, CAAS, www.cnsilkworm.com) and identified to be highly resistant to *Bombyx mori* nucleopolyhedrovirus (BmNPV) (Li et al. 2016). For better understanding of the mechanism of its antivirus, we analyzed the mitochondrial genome of *B. mori* BaiyuN. Total genomic DNA was extracted from the 3rd day of pupa and sequenced using the Illumina Miseq platform (Illumina lnc., San Diego, CA). A5-miseq v20150522 (Coil et al. 2015) and SPAdesv3.9.0 (Bankevich et al. 2012) software were used to assemble the obtained high quality paired-end reads. Finally, we got the complete mitochondrial genome of *B. mori* BaiyuN was 15,655 bp in length (GenBank accession no. MG797555), and included 13 protein-coding genes (PCGs), two rRNA genes (*rrnL* and *rrnS*), 22 tRNA genes, and a D-loop region (494 bp). The gene order and orientation of *B. mori* stain BaiyuN were similar to that of *B. mori* strains published (Zhang et al. 2016).

The nucleotide composition of *B. mori* strain BaiyuN was significantly biased (A, G, C, and T was 43.07%, 7.31%, 11.33%, and 38.29%, respectively) with A + T contents of 81.36%. The AT-skew and GC-skew of this genome were 0.059 and −0.216, respectively. Fourteen genes were transcribed on the J-strand included four PCGs (*ND1*, *ND4*, *ND4L*, *ND5*), two rRNAs, and eight tRNAs (*tRNA^Gln^*, *tRNA^Cys^*, *tRNA^Tyr^*, *tRNA^Phe^*, *tRNA^His^*, *tRNA^Pro^*, *tRNA^Leu^*, *tRNA^Val^*), whereas the others were oriented on the N-strand. Most of the protein-coding genes use ATN (N represents A, T, C, G) as the initiation codon whereas the *cox1* gene initiated with CGA, meanwhile, all the protein-coding genes ended with TAA.

To validate the phylogenetic position of *B. mori* BaiyuN, we used MEGA6 software (Tamura et al. 2013) to construct a maximum-likelihood tree (with 500 bootstrap replicates and Kimura 2-parameter model) containing complete mitogenomes of nine species (*B. mori*, *B. mandarina*, *Antheraea*, respectively, and *Drosophila melanogaster* as a outgroup control) derived from GenBank. The mitogenome sequence of *B. mori* BaiyuN is similar to the sequence published by Lee et al (GenBank accession no. AF149768). So, five strains (Aojuku, Backokjam, C108, BaiyuN, Xiafang) of *B. mori* were first clustered together in the ML phylogenetic tree (Figure 1). The mitochondrial DNA of *B. mori* used in different geographic varieties and different tissues suggesting that the mitochondrial DNA of *B. mori* are may very conservative.

**Figure 1.**
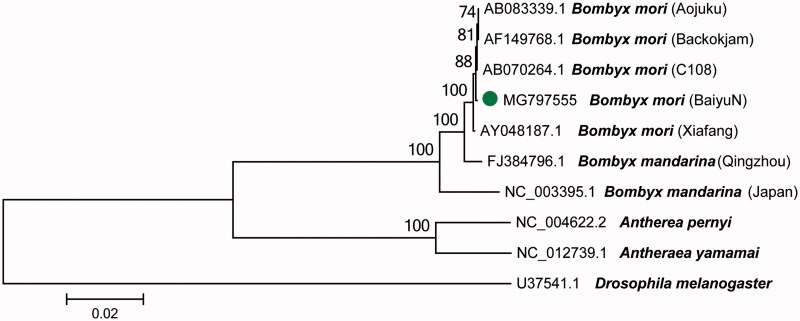
A maximum-likelihood tree illustrating the phylogenetic position of *B. mori* BaiyuN among other *Bombyx* species. The Maximum-likelihood analysis was conducted using the complete mitogenomes, and numbers at each node are bootstrap probabilities by 500 replications shown only when they are 50% or larger. GenBank accession numbers of mitogenomic sequences for each taxon are shown.
